# The Association between Periodontitis and Inflammatory Bowel Disease: A Systematic Review and Meta-analysis

**DOI:** 10.1155/2021/6692420

**Published:** 2021-03-12

**Authors:** Yangheng Zhang, Dan Qiao, Rixin Chen, Feng Zhu, Jianfeng Gong, Fuhua Yan

**Affiliations:** ^1^Department of Periodontology, Nanjing Stomatological Hospital, Medical School of Nanjing University, Nanjing 210008, China; ^2^Department of General Surgery, Jinling Hospital, Medical School of Nanjing University, Nanjing 210002, China

## Abstract

**Background:**

It has been reported that patients with inflammatory bowel disease (IBD) are more susceptible to periodontitis. However, data regarding the risk of periodontitis in IBD patients are scarce, and results from individual studies remain controversial. The aim of this study is to investigate the risk of periodontitis in IBD patients.

**Methods:**

Web of Science, PubMed, and Embase were searched for studies investigating the risk of periodontitis in the IBD patient population from Jan. 2000 to Nov. 2020. Articles were included if they contained the number of people with IBD diagnosed with periodontitis (or periodontal disease parameters) compared with a control group. Case reports, reviews, animal studies, and articles without available abstracts were excluded. A pooled odds ratio (OR) and 95% confidence interval (CI) were calculated to assess the association between periodontitis and IBD.

**Results:**

Six studies were included in the meta-analysis. The overall risk of periodontitis was significantly higher in IBD patients than controls (OR: 2.10, 95% CI: 1.60-2.74; *I*^2^ = 27%). In particular, Crohn's disease (CD) and ulcerative colitis (UC) were both linked to an increased risk of periodontitis (OR: 1.72, 95% CI: 1.36-2.19; *I*^2^ = 0% for CD vs. OR:2.39, 95% CI: 1.19-4.80; *I*^2^ = 85% for UC).

**Conclusions:**

IBD patients are at higher risk of periodontitis than controls. After subgroup analysis, the elevated risk remained significant when analyzing CD or UC alone. UC patients were at higher risk of developing periodontitis than CD patients.

## 1. Introduction

Inflammatory bowel disease (IBD) is a chronic inflammatory disorder of the gut, which includes Crohn's disease (CD) and ulcerative colitis (UC) [1]. UC mainly affects the colon and rectum, with inflammation restricted to the mucosal layer [2], whereas CD affects the entire gastrointestinal tract and involves transmural inflammation [2]. Although both diseases are characterized by inflammation of the gut, certain extraintestinal manifestations could occur in the wake of systemic inflammation triggered by the disease [3, 4]. Although the pathogenesis of IBD remains unknown, disturbed host-microbiota interactions and aberrant activation of the host immune system are thought to be critical factors [5].

Up to 9% of patients with IBD present with oral manifestations [6, 7], of which periodontitis is closely related to IBD pathogenesis. Periodontitis is a chronic inflammatory disease involving the supporting structures of the teeth [8]. Periodontitis is common worldwide, with prevalence ranging from 4% to 76% in developed countries and 50% to 90% in developing countries [9]. The pathogenesis of periodontitis mirrors that of IBD and mainly involves interactions between the host and oral pathogens. Consequently, the host inflammatory response against the pathogens leads to the destruction of soft and hard periodontal tissues [10].

Several studies have found that IBD is often associated with a higher prevalence of periodontitis. Brito et al. have shown that the prevalence of periodontitis is higher in patients with IBD than in healthy subjects [11]. In addition, periodontal lesions in IBD patients are more serious and extensive compared with those of control patients [12, 13]. In contrast, a case-control study argued that IBD was not associated with worsened periodontal conditions [14], indicating that IBD did not necessarily enhance susceptibility to periodontitis. Another study reported that poor oral hygiene, which is often linked to higher incidences of periodontitis, was inversely correlated with IBD [15]. Data about the prevalence of periodontitis in IBD patients are limited and controversial. Therefore, the aim of this meta-analysis was to systematically evaluate the risk of periodontitis in IBD patients following the PICO principle (P: human subjects; I: IBD; C: No IBD; O: periodontitis).

## 2. Materials and Methods

This meta-analysis was reported according to the instructions of the Preferred Reporting Items for Systematic Review and Meta-Analyses (PRISMA) statement [16] and conducted according to the Cochrane Handbook [17].

### 2.1. Search Strategy

Web of Science, PubMed, and Embase were extensively searched from Jan. 2000 to Nov. 2020. The following medical subject heading terms were used: “inflammatory bowel disease,” “Crohn's disease,” “ulcerative colitis,” “periodontal disease,” “periodontitis,” and “gingivitis.” All items were searched as key words or medical subject headings (MESH) where available. The electronic search strategy for PubMed, for example, was as follows: (“inflammatory bowel disease” or “Crohn's disease” or “ulcerative colitis”) and (“periodontal disease” or “periodontitis” or “gingivitis”). The search was performed in English. Based on titles and abstracts, the records were screened, and relevant studies were selected for full-text assessments. References of the eligible studies were also checked for studies not identified by the primary search strategy. The inclusion and exclusion criteria for published studies are shown in Table 1.

### 2.2. Data Extraction

A study was deemed eligible if it met all the inclusion criteria and none of the exclusion criteria. From each study enrolled, three authors (Y.Z., D.Q., and R.C.) extracted information related to the name of the journal, names of authors, year of publication, location of study, study design, study population, sample size, and periodontal manifestations. Disagreement or uncertainty was resolved by discussion among the authors. In these studies, periodontitis was assessed using different markers and indices, such as the plaque index, gingival index, bleeding on probing, pocket depth, clinical attachment loss, gingival recession, and periodontal index. If the information provided in the study was insufficient, the corresponding author of the article was contacted for the missing data. However, all studies provided sufficient information about outcomes.

### 2.3. Quality Assessment

The Newcastle-Ottawa Scale (range, 0–9 stars) was used to assess the quality of the enrolled studies [18]. Briefly, a maximum of 9 stars were given after comprehensive evaluation on 9 aspects (e.g., selection of cases and controls, comparability, and outcomes). Studies possessing 5 or more stars were deemed as moderate or high methodological quality.

### 2.4. Statistical Analysis

The association between periodontitis and IBD was calculated using odds ratios (ORs) extracted from individual studies. A random-effects model was used to obtain the pooled ORs with the 95% confidence interval (CI). Heterogeneity was evaluated using the Cochrane *I*^2^ statistic, with *I*^2^ > 50% indicating substantial heterogeneity [19]. Subgroup analyses were performed in the CD and UC groups. Sensitivity analyses were conducted by sequential removal of single studies to investigate if a single study was driving the results. Statistical analysis was conducted using the R packages meta [20] and metafor (version 3.6.3; Linux; R Core Team) [21]. A two-sided *p* value < 0.05 was considered statistically significant.

## 3. Results

### 3.1. Study Characteristics

**Figure 1:**
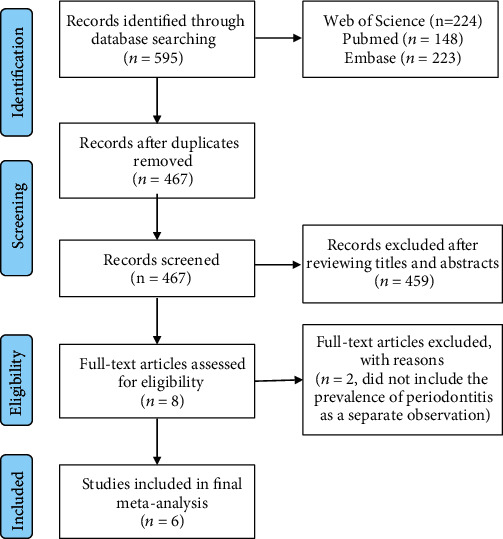
Flow chart demonstrating the study selection process.

The literature search process is summarized in Figure 1. Briefly, 467 articles were retrieved by an initial database search, including exclusion of duplications. Four hundred and fifty-nine publications were excluded after screening the abstracts. Two relevant publications were excluded because they did not include the prevalence of periodontitis as a separate observation [7, 22]. Finally, a total of 6 publications were pooled for analysis with a total of 3711 patients [11, 12, 14, 23–25]. The included studies were published between 2004 and 2020, reporting data from Greece, Germany, Brazil, Sweden, Jordan, and China. The characteristics of these studies are shown in Table 2.

### 3.2. Study Quality

All 6 studies ranked between 7 and 9 stars according to the Newcastle-Ottawa Scale (Table 3); they were all of reasonably moderate or high quality with clear definitions of cases, representativeness of the cases, and comparability based on design or analysis.

### 3.3. Overall Risk of Periodontitis

**Figure 2:**
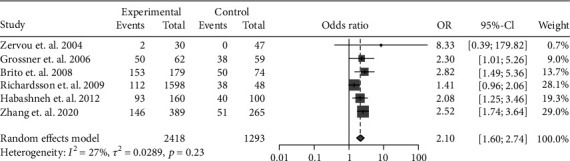
Forest plot demonstrating the association between the risk of periodontitis and IBD (*p* < 0.001).

**Figure 3:**
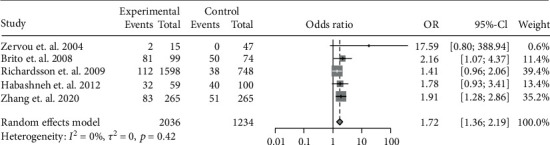
Forest plot demonstrating the association between the risk of periodontitis and CD (*p* < 0.001).

**Figure 4:**
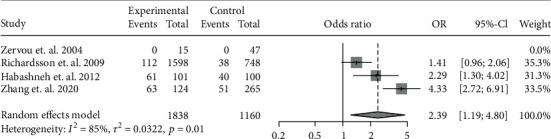
Forest plot demonstrating the association between the risk of periodontitis and UC (*p* = 0.0145).

The data of the 6 studies were pooled to assess the influence of IBD diagnosis on the development of periodontitis. In all, 556 cases of periodontitis were identified in 2418 IBD patients, while 217 cases were identified in 1293 controls. IBD was associated with a 2.10-fold risk for periodontitis (OR: 2.10, 95% CI: 1.60-2.74; *I*^2^ = 27%) (Figure 2). Because CD and UC involve different pathogeneses and disease behaviors, we further investigated the risk of periodontitis in CD and UC separately. In particular, 5 studies had accessible data on CD-related periodontitis and 4 had data on UC-related periodontitis. A pooled analysis showed the OR of periodontitis for CD patients was 1.72 (95% CI: 1.36-2.19; *I*^2^ = 0%) (Figure 3), whereas that for UC patients was 2.39 (95% CI: 1.19-4.80; *I*^2^ = 85%) (Figure 4).

### 3.4. Sensitivity Analysis

**Figure 5:**
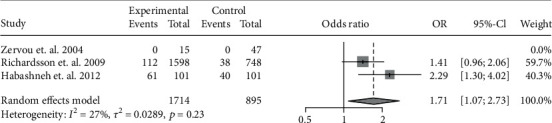
Forest plot demonstrating the association between the risk of periodontitis and UC after excluding the study conducted by Zhang et al. [25] (*p* = 0.0239).

Heterogeneity analysis showed that the *I*^2^ statistic was highest when analyzing the UC subgroup (*I*^2^ = 27%; *p* = 0.23 for IBD vs. *I*^2^ = 0%; *p* = 0.42 for CD and *I*^2^ = 85%; *p* < 0.01 for UC). The potential effects of any single study on heterogeneity were investigated by sensitivity analysis. Briefly, each study was removed sequentially to obtain the OR. When analyzing the remaining studies, we found that the heterogeneity across studies significantly decreased after removing the study by Zhang et al. [25] (*I*^2^ = 49%, *p* = 0.16), suggesting it was the source of the heterogeneity. The OR of periodontitis for UC after exclusion of the Zhang et al. study was 1.71 (95% CI: 1.07-2.73; *I*^2^ = 49%) (Figure 5).

## 4. Discussion

In recent decades, the association between IBD and periodontitis has been recognized on account of their similar etiologies. Both diseases involve dysbiotic microbiota, deregulation of the immune response, and chronic inflammation in genetically susceptible individuals [26–28]. Our study found that IBD patients had a higher risk of periodontitis than controls (OR: 2.10, 95% CI: 1.60-2.74; *I*^2^ = 27%), which was in agreement with previous publications [29–32]. Notably, the OR was higher in the UC subgroup than in the CD subgroup according to our analysis (OR: 1.72, 95% CI: 1.36-2.19; *I*^2^ = 0% for CD vs. OR: 2.39, 95% CI: 1.19-4.80; *I*^2^ = 85% for UC).

The number of publications included in the current meta-analysis is relatively small because of limited relevant research and the strict inclusion criteria. Because this study is aimed at calculating the risk of periodontitis in the IBD population, only publications with clear diagnosis of periodontitis were selected. Two studies that investigated the community periodontal index of treatment needs (CPITN) and loss of attachment at sites with maximal periodontal pocket depth (LA-PPD) were not included in this study [7, 22]. Despite the small number of eligible publications, this meta-analysis included 2418 cases of IBD and 1293 controls; overall, they indicated a higher risk of periodontitis in IBD patients than controls.

Microbiota play important roles in the pathogenesis of IBD and periodontitis [33]. A dysbiotic microbial community initiates nonresolving, chronic inflammation, leading to disruption of periodontal tissue or intestinal mucosa. Previous studies have shown significant differences in salivary microbiota compositions between IBD patients and controls [34]. It was found that overall diversity decreased significantly in the oral microbiome of pediatric CD patients [35]. Lira-Junior et al. suggested that certain species might damage host-microbe interactions in patients with untreated periodontal disease and IBD [36]. In a recent study, a distinct saliva microbiota dysbiosis in IBD was observed using 16S rRNA gene sequencing [37]. The results showed that some oral biofilm-forming bacteria, including Absconditabacteria (SR1), Saccharibacteria (TM7), *Leptotrichia*, *Prevotella*, *Bulleidia*, and *Atopobium*, were significantly increased [37]. However, the subgingival microbiota in IBD, which are closely related to periodontitis, are less well characterized. Periodontitis could lead to dysbiotic oral microbiota and potentially alter the gut microbiota [38]. Every day, more than 10^12^ oral bacteria in swallowed saliva can enter the gut and affect the gut's microbial composition [39, 40], which subsequently decreases the expression of tight-junction proteins and increases gut bacterial translocation and systemic inflammation [41]. Future large-sample studies using in-depth sequencing techniques are warranted to delineate the microbial link between IBD and periodontitis.

The aberrant immune response during IBD could cause inflammation of the oral cavity. IBD is an autoimmune disease, whereas poor oral health is associated with an overly aggressive immune response in local periodontal tissues [42]. Elevated cytokines may be released systemically in the processes of IBD. Figueredo et al. reported that higher IL-18 levels were detected in serum from patients with IBD and periodontitis [43]. In addition, increased levels of proinflammatory cytokines have been found in saliva from IBD patients. Higher levels of salivary TNF-*α*, IL-1*β*, and IL-6 were found in patients with active CD, and elevated salivary TNF-*α* and IL-6 correlate with specific oral lesions [44]. TNF inhibitors have been used to treat IBD and could reduce inflammation and stop disease progression [45]. Similarly, anti-TNF treatment has shown promising results in periodontitis. In periodontitis animal models, anti-TNF treatment can reduce inflammatory cell recruitment and bone loss [46, 47]. This evidence indicates that IBD and periodontitis share similar immunological etiologies.

Despite their similar etiologies, it is likely that IBD and periodontitis could trigger one another. That is, periodontitis, as one of the extraintestinal manifestations of IBD, could present before or after the onset of intestinal symptoms. There were limited studies that evaluated the risk of IBD in patients with periodontitis [48, 49]. A cohort study reported a 1.56-fold significantly higher risk of UC, but not CD, in patients with periodontal disease [48]. Similarly, it was found that the risk of developing UC increased significantly in patients with periodontitis in a recent retrospective study involving 1 million subjects [49]. In this meta-analysis, it was found that patients with UC had a higher risk for developing periodontitis than CD patients (OR:2.39 vs. OR: 1.72). This evidence suggests periodontitis is more correlated with UC than with CD.

Certain limitations must be considered when interpreting the results of this study. First, there were some differences in the definition of periodontitis in the included studies, which may have caused some bias. Furthermore, the use of studies including self-reported periodontitis could have introduced measurement error. The risk of developing periodontitis in IBD subjects may be higher in fact. Second, the risk of developing periodontitis among patients with IBD was not adjusted for relevant factors, especially medications and smoking habits. The use of antibiotics, immunomodulatory drugs, and corticosteroids are possible confounders for evaluating the risk of periodontitis in IBD patients. Smoking is a risk for periodontitis [50], whereas individuals who smoke have a higher risk of CD but a lower risk of UC [51]. Smoking habits could influence the development of both periodontitis and IBD. Third, all the included studies were case-control studies. Well-designed prospective cohort studies of patients with/without IBD and periodontitis are needed to determine the causal relationship. Lastly, publication bias was not evaluated by funnel plots because the number of included studies was too small.

## 5. Conclusions

This meta-analysis showed that IBD patients are at higher risk of developing periodontitis than controls. After subgroup analysis, the increased risk remained significant when analyzing CD or UC alone. UC patients were at higher risk of developing periodontitis than CD patients. Additional large-scale, prospective studies incorporating professional dental care and IBD centers are essential to clarify the relationship between periodontitis and IBD.

## Figures and Tables

**Table 1:** Inclusion and exclusion criteria for published studies.

Inclusion criteria	Exclusion criteria
a) Studies on human subjects b) Cohort or case-control studies including patients with known IBD diagnoses and no IBD c) Studies using periodontitis as a primary observation d) Studies reporting an estimated measure of effect size ([RR], [HR], or [OR]) and its associated 95% confidence interval (CI) or those providing calculable data	a) Case reports b) Reviews c) Animal studies d) Articles without available abstracts e) Duplicated studies

**Table 2:** Characteristics of the included studies.

Author, year of publication, and country	Study design	Study size	IBD patients	Control	Rate, IBD/control	Periodontal manifestations	Definition of periodontitis	Reference
Zervou et al. (2004), Greece	Case-control	77	30	47	0.06/0	Periodontitis; gingivitis; gingival bleeding	Not described	[24]
Grössner et al. (2006), Germany	Case-control	121	62	59	0.81/0.64	%PI; %BOP; PPD; %CAL ≥ 4 mm; %CAL ≥ 5 mm	≥1 sites with CAL ≥ 4	[14]
Brito et al. (2008), Brazil	Case-control	253	179	74	0.85/0.67	Periodontitis (at least 4 × CAL ≥ 3 mm); %PI; %BOP; PPD; CAL; %CAL ≥ 3 mm	≥4 sites in different teeth with CAL ≥ 3 mm	[11]
Rikardsson et al. (2009), Sweden	Case-control	2346	1598	748	0.7/0.51	Periodontitis; gingival bleeding	Self-reported	[23]
Habashneh et al. (2012), Jordan	Case-control	260	160	100	0.58/0.4	Periodontitis; PI; GI; PPD; CAL; GR; %BOP; %PPD ≥ 3; %PPD ≥ 4; %CAL ≥ 3; %CAL ≥ 4; %CAL ≥ 5	≥4 teeth with one site or more having PD ≥ 4 mm and CAL ≥ 3 mm	[12]
Zhang et al. (2020), China	Case-control	654	389	265	0.38/0.19	PD; CAL; GR; GI; PI; %PD ≥ 4; %PD ≥ 5; %CAL ≥ 3; %CAL ≥ 4; %GR ≥ 1; %GR ≥ 2; %BOP; %CI	≥2 interproximal sites with CAL ≥ 3 mm, and ≥2 interproximal sites with PD ≥ 4 mm (not on the same tooth), or ≥1 site with PD ≥ 5 mm	[25]

PI: plaque index; BOP: bleeding on probing; PPD: probing pocket depth; CAL: clinical attachment loss; GI: gingival index; GR: gingival recession; CI: calculus index.

**Table 3:** Methodological quality of case-control studies included in the meta-analysis.

Study	Selection				Comparability	Outcomes			Total scores
	Case definition adequate	Representative-ness of the cases	Selection of controls	Definition of controls	Comparability based on design or analysis	Ascertainment of exposure	Same method of ascertainment for cases and controls	Nonresponse rate	
Zervou et al. (2004) [24]	☆	☆		☆	☆	☆	☆	☆	7
Grössner et al. (2006) [14]	☆	☆	☆	☆	☆☆	☆	☆	☆	9
Brito et al. (2008) [11]	☆	☆		☆	☆☆	☆	☆	☆	8
Rikardsson et al. (2009) [23]	☆	☆	☆	☆	☆		☆		6
Habashneh et al. (2012) [12]	☆	☆		☆	☆☆	☆	☆		7
Zhang et al. (2020) [25]	☆	☆		☆	☆☆	☆	☆	☆	8

## Data Availability

The datasets generated or analyzed during the current study are available from the corresponding author on reasonable request.
